# Novel advances in strategies and applications of artificial articular cartilage

**DOI:** 10.3389/fbioe.2022.987999

**Published:** 2022-08-22

**Authors:** Yifei Chen, Chenyue Zhang, Shiyong Zhang, Hexu Qi, Donghui Zhang, Yifei Li, Jie Fang

**Affiliations:** ^1^ State Key Laboratory of Oral Diseases & National Clinical Research Center for Oral Diseases & Dept. of Orthodontics, West China Hospital of Stomatology, Sichuan University, Chengdu, China; ^2^ State Key Laboratory of Biocatalysis and Enzyme Engineering, School of Life Science, Hubei University, Wuhan, China; ^3^ Key Laboratory of Birth Defects and Related Diseases of Women and Children of MOE, Department of Pediatrics, West China Second University Hospital, Sichuan University, Chengdu, China

**Keywords:** bioengineering, artificial articular cartilage, electrospinning, 3D printing, self-assembly

## Abstract

Artificial articular cartilage (AC) is extensively applied in the repair and regeneration of cartilage which lacks self-regeneration capacity because of its avascular and low-cellularity nature. With advances in tissue engineering, bioengineering techniques for artificial AC construction have been increasing and maturing gradually. In this review, we elaborated on the advances of biological scaffold technologies in artificial AC including freeze-drying, electrospinning, 3D bioprinting and decellularized, and scaffold-free methods such as self-assembly and cell sheet. In the following, several successful applications of artificial AC built by scaffold and scaffold-free techniques are introduced to demonstrate the clinical application value of artificial AC.

## Introduction

Articular cartilage (AC) is a connective tissue covered on the ends of bones in an articulating joint. It provides a low-friction surface to facilitate movements of the joints and serves as the load-bearing part which absorbs and distributes loads applied to the joint to protect the subchondral bones. AC tissue is mainly composed of water, chondrocytes, predominantly type II collagen, and proteoglycans (Mobasheri et al., 2008). However, AC can be easily damaged by traumatic injuries to the joint, aging, or diseases. Due to its avascular and low-cellularity nature, AC has very limited self-healing capacity. Hence, articular cartilage lesions become almost unrecoverable. If damages to AC were not treated properly, cartilage defeat will lead to progressive degeneration and destruction of the cartilage and ultimately result in arthritis, usually osteoarthritis (OA) in most cases (Giunta et al., 2015; Musumeci et al., 2015).

Notwithstanding various surgical approaches have been introduced in clinics and some of them had receive positive therapeutic outcomes, current surgical strategies are designed to treat small cartilage defects, but they lack long-term durable and sufficient clinical outcomes (Kwon et al., 2019). Therefore, cartilage tissue engineering techniques are a promising alternative treatment strategy. Artificial AC is a prosthesis designated by tissue engineering for cartilage repair, combining biomaterials, seed cells, growth factors and biochemical, biophysical, and biomechanical stimuli (Kwon et al., 2019). Those seed cells including chondrocytes, chondroprogenitor cells and different kinds of stem cells derived from bone marrow, adipose tissue, embryo, umbilical cord, etc are required to have differentiation potential and chondrogenic ability (Bai et al., 2022). Cartilage tissue engineering aims at regenerating chondral grafts to restore structure–function properties in degenerated cartilage. The construction of artificial AC can be divided into two categories according to the use of cell scaffolds. The first category is the methods using cell scaffolds, in which seed cells and growth factors are loaded onto artificially constructed three-dimensional cell scaffolds to construct cell-material complexes and then implanted into the body (Salerno et al., 2019). The second category is the techniques without scaffolds, which use cells only to construct implants or directly implant them into the defect site to construct artificial AC without the intervention of scaffold materials (De Pieri et al., 2021).

**FIGURE 1:**
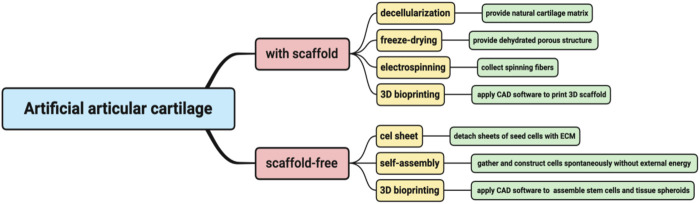
Brief introduction of the tissue engineering techniques for artificial AC.

In this review, we highlighted several mature techniques including scaffold-based and scaffold-free approaches to constructing artificial, presented the applications of artificial AC formed by different techniques and compared their advantages and disadvantages, aiming to provide a reference for the application of artificial articular cartilage in the repair treatment of the defect in knee, hip, temporomandibular joint (TMJ) and other joints (Figure 1).

## Technologies for artificial AC with scaffold

### Decellularization

Decellularized cartilage matrix from cartilage is an ideal natural scaffold for artificial AC tissue engineering. However, traditional decellularized techniques such as physical, chemical, and enzymatic treatments can decellularize the cartilage matrix (Luo et al., 2019), but all have certain impurity residues and damage to the scaffold structure. Therefore, some new techniques are explored to overcome the defect of the old ones. To reduce the harmful physical and chemical steps and the duration of the decellularized process, a perfusion-based bioreactor (PBB) method was developed to decellularize bovine ACs efficiently. The PBB method includes channelizing and placing cartilages into perfusion bioreactor and the structure and components of the extracellular matrix (ECM) remained relatively intact with high porosity and appropriate compressive property which supported the viability and proliferation of human chondrocytes. All the results display PBB technique can accelerate and improve the decellularization of cartilages for cartilage repair. (Mousavi et al., 2021). Moreover, supercritical carbon dioxide (SCCO2) extraction technology which leaves no chemical solvent residue, solubilizes, removes oil and lipids, and can sterilize and disinfect has become a good choice to eliminate impurity damage and create biocompatible decellularized scaffolds (Chen et al., 2021). In another study, decellularized allogeneic and xenogeneic AC matrix scaffolds modified by laser (LM-CMS) enhanced the degree of decellularization and were conducive to cell adhesion thanks to high porosity and micropores when compared with the initial CMS, exhibiting excellent cartilage regeneration ability of LM-CMS(Li et al., 2019b).

### Freeze-drying

Freeze-drying is a traditional method to manufacture porous scaffolds for tissue engineering and biological applications. In this method, the water in the material is first converted into ice at a low temperature, then the ice is sublimated by a vacuum environment to remove water before the dehydrated porous structure is obtained (Piazza et al., 2020). Moreover, the pore diameter and porosity can be controlled by changing the freezing method (Cardea, 2021). This method can retain the physical and chemical properties of the material and prepare scaffolds with high porosity, providing a larger specific surface area that contributes to seed cell colonization, metabolism and proliferation (Valencia et al., 2018; Mirtaghavi et al., 2020). When constructing cartilage scaffolds, this technique can be adopted by a wide range of biomaterials. Bahrami et al. (2019) successfully constructed a three-dimensional scaffold for AC regeneration by the freeze-drying method using alginate, a natural material similar to the extracellular matrix with superior porosity, degradation rate, and mechanical. On this scaffold, periodontal ligament stem cells (PDLSCs) successfully differentiated into osteoblast-like cells, indicating the scaffold has excellent osteogenic properties, as well. In another study (Klimek et al., 2022), the freeze-drying technique optimized the porosity ratio and specific surface area of a novel scaffold made of curdlan/whey protein isolate-based biomaterial, promoting the viability and proliferation of the chondrocytes. Compared with natural materials, this scaffold showed more suitable and controllable degradation rate. Although freeze-drying can promote AC formation, it consumes more energy and costs more (Zhao et al., 2018). Additionally, while the porosity of the scaffolds is increased by freeze-drying, the mechanical properties of some materials are reduced. When necessary, the mechanical properties of the s scaffold should be improved in combination with chemical cross-linking, electrospinning and other methods. (Liu et al., 2015; Oh et al., 2018; Wu et al., 2019).

### Electrospinning

Electrospinning is a manufacturing process broadly applied in tissue engineering, textile industry, energy harvesting, etc. (Chen et al., 2020c), which is capable to construct biomimetic nanofiber scaffolds for artificial AC. This technology mainly includes four steps (Badrossamay et al., 2014): 1) drives the polymer solution to the needle through the injection pump to form a droplet. 2) The spherical droplet overcomes the surface tension of the solution under the action of the electric field to form a conical Taylor cone. 3) As the current intensity gradually increases to the critical value, the electric field force will overcome the surface tension of the liquid and eject the charged micro-jet from the Taylor cone. 4) The micro-jet is pulled by the electric field force in the electric field, and as the solvent volatilizes, the spinning fiber is formed at the receiving end, and finally solidified into fiber. Electrospinning can apply to an extensive range of scaffold materials for artificial cartilage.

In a previous study (Menezes and Arinzeh, 2020), the gelatin-based scaffolds containing partially sulfated cellulose (pSC) were electrospun at a flow rate of 6.5 ml/h under 40 kV. With porous morphology, it is beneficial to the chondrogenic differentiation of human mesenchymal stem cells (hMSCs). However, traditional electrospinning uses a flat plate to collect the fibers, thus can only produce two-dimensional fiber membranes. In consequence, cartilage scaffolds are assembled by tightly stacked membranes, which hinders the infiltration of cells into the scaffold. To solve this problem, except the assistance of other methods such as freeze-drying and self-assembly, improved electrospinning methods are introduced to build three-dimensional scaffolds. (Chen et al., 2016; Li et al., 2019a; Chen et al., 2020c). Additionally, to prolong the effect of growth factors, the core-shell structure and nano-coating formed by coaxial electrospinning can effectively control the release rate of growth factors and drugs inside the fibers (Rafiei et al., 2020). Polycaprolactone/glucosamine sulfate (PCL/GAS) scaffold formed by encapsulating GAS in PCL using coaxial electrospinning achieved slow release of GAS. The results showed that this novel structure obtained excellent cytocompatibility and mechanical properties and prolonged the stimulating effect of GAS on proteoglycan and collagen synthesis in the extracellular matrix of cartilage, demonstrating the prospect of coaxial electrospinning in the fabrication of cartilage scaffolds that is beneficial to the maintenance and sustained release of growth factors and drugs (Chen et al., 2020b).

However, due to the loss in the electrospinning process, only 40–50% of the growth factors loaded on the material can be released by the scaffold, resulting in a decrease in the efficiency of AC regeneration and an increase in manufacturing cost (Martin et al., 2021). Therefore, the appropriate storage of the cartilage scaffold with growth factor also plays an important role in chondrogenesis.

### 3D bioprinting

3D bioprinting is a technology derived from 3D printing that creates complex geometries from 3D digital models generated by computer-aided design (CAD) software, which allows precise regulation of the size and structure of biological scaffolds by printing according to a preset program (Xu et al., 2022). In the manufacture of cartilage scaffolds, this emerging technology enables the production of AC scaffolds with controlled structure and porosity, and the ability to precisely tune the geometry and mechanical properties of the scaffold (Dai et al., 2020). Extrusion printing forms cartilage scaffolds by extruding biomaterials and stacking them layer by layer according to a computer preset. In the practice of extrusion bioprinting cartilage scaffolds, this method has been found to build mechanically stronger and more accurate scaffolds and to promote chondrogenesis of seed cells seeded on the scaffold (Boularaoui et al., 2022; Mueller et al., 2022). Li et al. (2021) used silk fibroin and tyramine-substituted gelatin (SF-GT) to construct macro-porous hydrogel scaffolds by extrusion printing and compared the differentiation of seed cells seeded on the scaffolds *via* cell suspension and *via* cell aggregate. The results indicated that the former tended to present fibrochondrocyte phenotype, whereas the latter showed hyaline cartilage phenotype, suggesting that it may be possible to build different types of AC on an extrusion-bioprinted scaffold by changing the ways of cell seeding. Coaxial extrusion printing derived from extrusion printing enables direct printing of cell-loaded hydrogels by changing the hydrogels in the shell and the core, and can build the layered structure of cartilage, namely, transparent cartilage and calcified cartilage, to better mimic the inhomogeneities and anisotropies of AC and promote Osteochondral tissue regeneration (Mow and Guo, 2002; Idaszek et al., 2019). However, extrusion printing is relatively less accurate in 3D bioprinting and the shear force generated in this way may affect cell survival in bioinks with higher cell density (Miri et al., 2019). Stereolithography (SLA) is a faster and more accurate 3D printing technique that uses a laser of a specific wavelength and intensity to focus on the surface of light curing material to make it light curing, and the layers are stacked to form a three-dimensional entity (Grigoryan et al., 2021; Zhang et al., 2021). Aisenbrey et al. (Aisenbrey et al., 2018). Developed a novel hybrid scaffold combing support resin structure printed by SLA with an injectable and photopolymerized hydrogel which is infilled into it to incorporate cartilage cells. This hybrid scaffold provides mechanical support while loading autologous chondrocytes and hydrogel matrix, which enables independent design of the support structure and the hydrogel such that in the future the hydrogel can be designed to degrade rapidly while the support structure can be designed to degrade slowly for maintaining the health of cells embedded in the scaffold. Schoonraad et al. (2021) also constructed a hybrid cartilage scaffold by digital light processing-based SLA, which can isolate the mechanical and biochemical needs and fabricating multi-layered structures through the use of light curing resin to mimic osteochondral interface.

## Technologies for scaffold-free artificial AC

### Cell sheet

Cell sheet containing seed cells and extracellular matrix (ECM) is obtained by culturing stem cells and transplanted into the defective area after detached with external force to promote tissue recovery through gradual differentiation and proliferation of the stem cell tissue. Cell sheet technology maintains cell functionality as scaffold-free constructs by ECM while enabling direct cell transplantation from *in vitro* culture to targeted sites *in vivo* (Kim et al., 2020). Thorp et al. (2020) used hBMSCs as allogeneic cell sources and cultured them on temperature-responsive culture dishes. By changing the temperature, the cell sheet contracted and detached to form the scaffold-free 3D MSC cellular constructs. After chondrogenic induction with chondrogenic media for 3 weeks, the 3D sheet *in vitro* differentiated to hyaline-like cartilage phenotypes. By direct adhesion of sheets to target tissue post-differentiation, the hyaline-like cartilage constructs from MSCs may be applied to future transplantable AC regeneration therapies.

### Self-assembly

In bioengineering, self-assembly is regarded as one of the promising tissue-engineering techniques in which the basic structural units in a material spontaneously form an ordered, stable and regular structure under the interaction of non-covalent bonds (Wang and Zhang, 2019). In scaffold-free AC engineering, self-assembly is required to gather and construct seed cells or tissue spheroids with mechanical (e.g., differential interfacial tension, differential adhesion, etc.) and/or biochemical stimuli without the effects of external energy which may increase the risk of cell damage (Lee et al., 2017). Generally, self-assembly depends on high cell density and is mainly mediated by N-cadherin that results in cell binding (Lee et al., 2017). Custom-made agarose molds that allowed for size and shape-specific constructs were utilized to self-assemble chondrocytes at high density after three-dimensional spherical suspension culture in a scaffold-free environment (Vapniarsky et al., 2022). Moreover, with biochemical stimuli (TGFb-1, C-ABC, LOX-L2) and mechanical stimulation, the biomechanical properties of the self-assembled neocartilages were improved (Vapniarsky et al., 2022).

### 3D bioprinting

Additionally, 3D bioprinting can also be applied in assembling stem cells and tissue spheroids to precisely control and maintain a scaffold-free construct shape. Ayan et al. (2020) printed hMSC spheroids by precisely positioning them in self-healing yield-stress gels which can keep the printed structure suspended and stable after printing, enabling free-shape cartilage structures according to the predefined program and self-assembly of tissue spheroids. 3D bioprinting system with needle arrays succeeded in producing vessel-like structures when forming 3D cellular structures using multicellular spheroids, which benefits the compact and orderly arrangement of cell spheroid and the biomechanical properties of the constructs. Grogan et al. (Machino et al., 2019) 3 days printed human embryonic-derived mesenchymal stem cell or infrapatellar fat pad mesenchymal stem cell microspheroids of 500 μm in diameter on microneedle arrays in a predefined arrangement. As a result, the two kinds of microspheroids fused into a tissue construct and completed cartilage regeneration successfully.

## Application of artificial AC

### Application of artificial AC with scaffold structure

**FIGURE 2:**
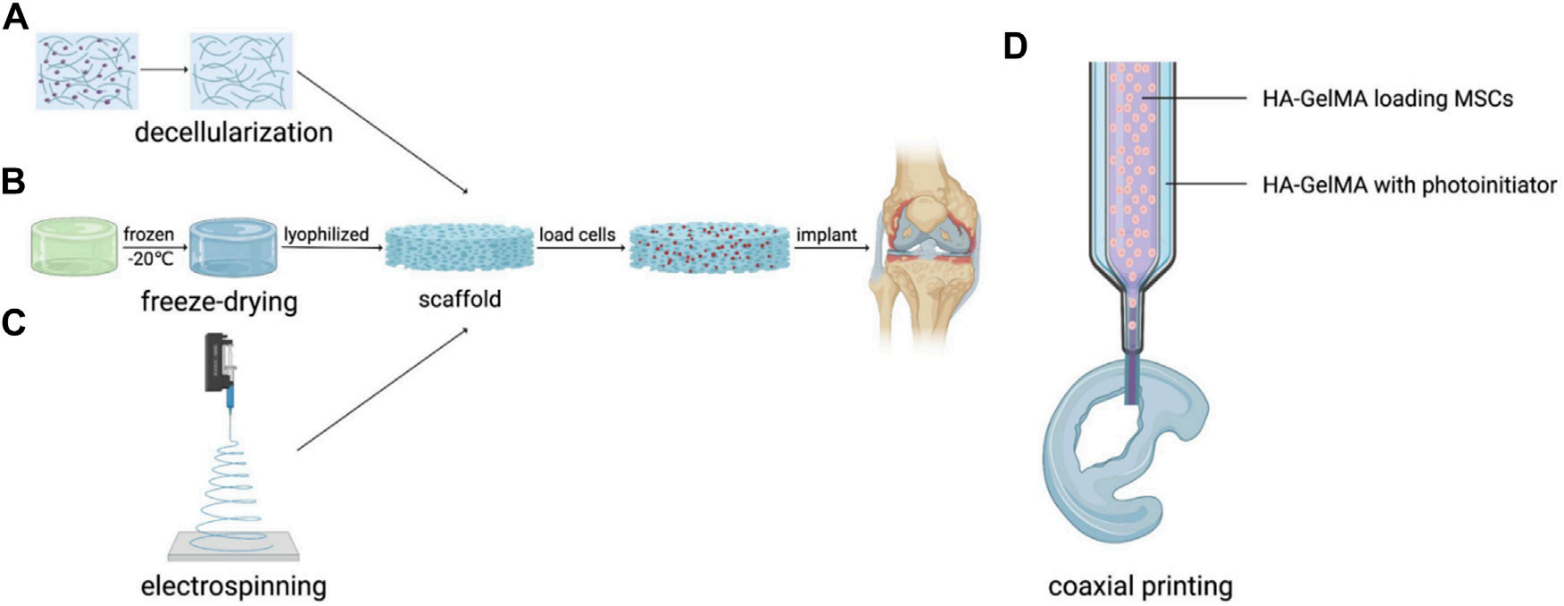
Application of artificial AC with scaffold structure. **(A)** Decellularization, **(B)** Freeze-drying, **(C)** Electrospinning and **(D)** Coaxial printing.

The combination of seed cells with scaffolds manufactured by the methods mentioned above provides a prospective strategy for AC regeneration in the clinic (Figure 2). The laser-modified decellularized cartilage matrix scaffold (LM-CMS) with micropores loading rabbits-derived chondrocytes was transported into rabbit knee articular cartilage defects after 8-weeks culture *in vitro* and resulted in neocartilage and better structural restoration, which also combines the virtues of high fidelity and good mechanical properties (Li et al., 2019b). Yu et al. (2021) constructed a porous scaffold with poly (L-glutamic acid)-graft-poly (ε-caprolactone)-poly (ethylene glycol) (PLGA-g-PCL-PEG) by freeze-drying method and the scaffold loading goat BMSCs (gBMSCs) was transplanted into the TMJ condyle defects of goats. After 2 months, the defects were totally healed and mimic the hierarchical structure of TMJ with ordered fibrocartilage and hyaline cartilage. Martin et al. (2021) added Transforming Growth Factor-β3 (TGF-β3) into the electrospinning solution to construct fibrous HA scaffold for chrongenesis, which was proved to be a feasible way to deliver biofactors and heal full-thickness porcine cartilage defects. To facilitate clinical application, Di Bella et al. (2018) explored *in situ* handheld printing for cartilage regeneration. They used gelatin methacrylamide and hyaluronic acid methacrylate hydrogel (HA-GelMA) loading MSCs as core and HA-GelMA bioink with photoinitiator as shell to coaxial print scaffold in the cartilage defects of sheep *in situ*, realizing the simultaneous coaxial extrusion of bioscaffold and cultured cells directly into the cartilage defect *in vivo* in a single-session surgery (Di Bella et al., 2018). If this *in situ* coaxial extrusion printing device is used in the clinic, there is no need for prior biomanufacturing of cartilage implants in the laboratory (Duchi et al., 2020).

### Application of artificial AC with scaffold-free strategy

**FIGURE 3:**
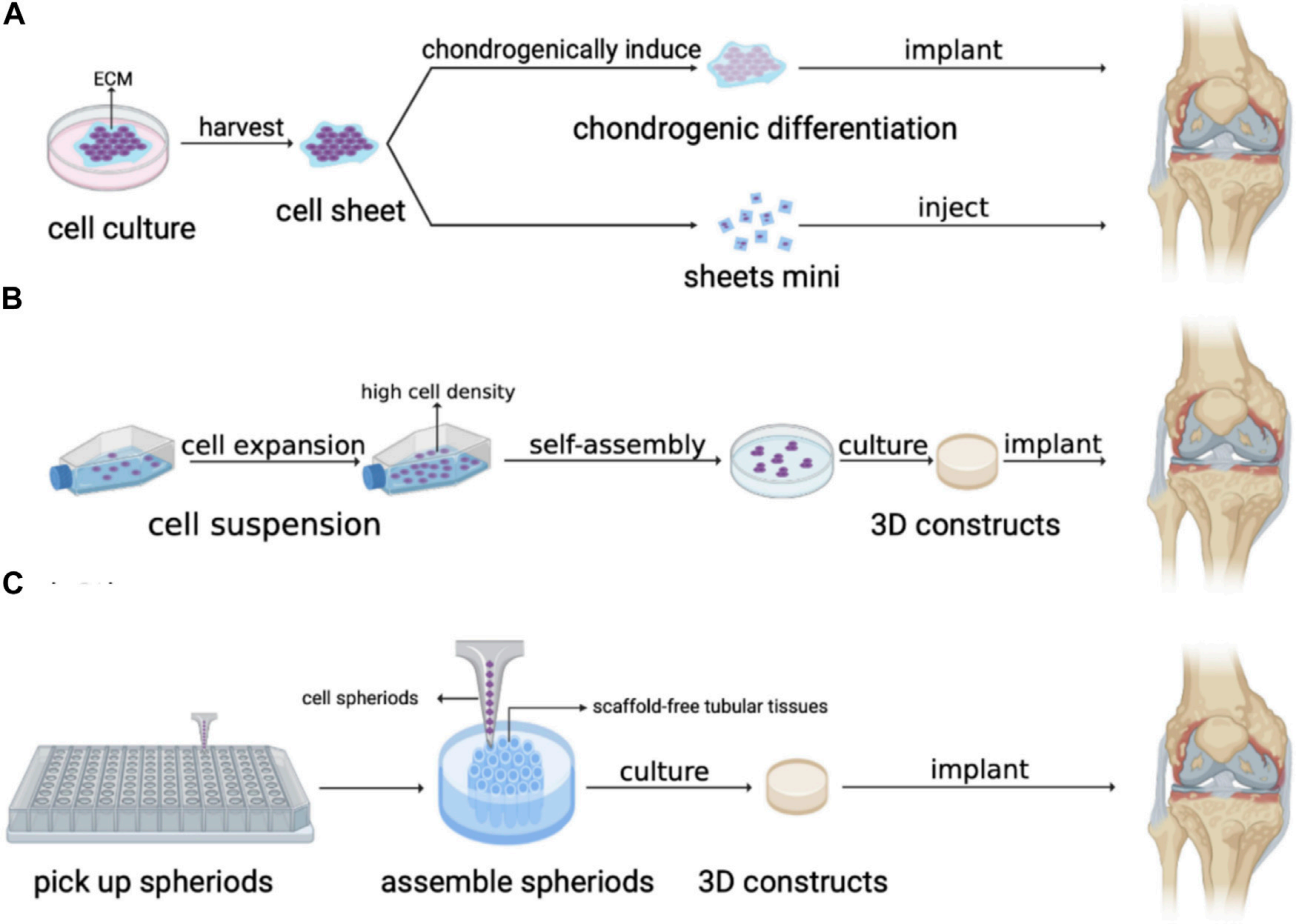
Application of artificial AC without scaffold structure. **(A)** Cell sheet, **(B)** Self-assembly. and **(C)** 3D bioprinting by needle array.

Scaffold-free artificial AC also has plenty of extensive applications in the repair of different articular (Figure 3). hAMSCs isolated from human amniotic membrane of placentas were cultured in cell sheet induction medium to fabricate chondrogenically induced hAMSC sheet which was proved to repair osteochondral defects successfully after being implanted into rabbits’ knee joints (Zou et al., 2022). Moreover, the cell sheet can be injected into the corresponding part to repair cartilage and avoid highly invasive surgery. Wasai et al. (2021) have developed injectable fragments of allogeneic polydactyly-derived chondrocyte sheets (PD sheets-mini), which resemble the characteristic of PD sheets and can maintain the cell viability of MCSs regardless of the clinically relevant needle gauge size to promote cartilage regenerations. Moreover, the safety of human juvenile cartilage-derived-chondrocyte sheets was proven in nude rat focal osteochondral defect models and the transplantation of chondrocyte sheets into human knee has shown efficacy in combination therapy for OA, which support the feasibility of cell sheet technique in clinical cartilage repair (Sato et al., 2019; Kondo et al., 2021). In TMJ regeneration, Vapniarsky et al. (2018) formed TMJ implants using allogeneic costal chondrocytes in conjunction with a scaffold-free, self-assembling process and implanted the constructs into the artificial defects in the minipig disc’s posterolateral portion. The results showed that the implants displayed mechanical properties similar to the native disc, which allowed for immediate load bearing and joint movement upon implantation, suggesting the successful regeneration of TMJ by the scaffold-free method. 3D printed scaffold-free artificial AC also obtains broad prospects for cartilage repair. Yamasaki et al. (2019) implanted scaffold-free constructs composed of autologous adipose tissue-derived mesenchymal stem cell (AT-MSC) spheroids by 3D bioprinting with needle-array into the osteochondral defects in the knees of mini-pigs. The results demonstrated that the scaffold-free AT-MSC can significantly help osteochondral regeneration in pigs (Yamasaki et al., 2019).

## Conclusion

In summary, the manufacturing methods of artificial cartilage with and without scaffolds have significant progress and broad prospects for clinical transformation, but these two methods also have their respective merits and demerits.

The AC tissue engineering using scaffold is realized mainly by seed cells, growth factors and scaffold materials. In cartilage tissue engineering, chondrocytes and mesenchymal stem cells (MSCs) such as bone marrow mesenchymal stem cells (BMSCs), adipose mesenchymal stem cells, etc. are widely used as seed cells because of their strong proliferative ability (Theodoridis et al., 2022). To promote the proliferation and differentiation of seed cells, growth factors are applied in the cell scaffolds, as well (Chen et al., 2020a). The ideal scaffold is required to have excellent biocompatibility, biodegradability, biocompatibility, appropriate pore size and structure for cell colonization, good mechanical strength, and a three-dimensional network structure (Zhang et al., 2019). In addition, it can also transport seed cells, growth factors, drugs, nutrients, metabolites, etc., and promote cell proliferation and differentiation at the meanwhile (Hastar et al., 2017). However, the degradation properties of some non-natural materials and toxicity of degradation products need to be tested in clinical studies.

The scaffold-free technique is mainly used to repair and regenerate cartilage by sending a mixture of seed cells and ECM or pure tissue spheroids into the site of AC damage and loss without forming an exogenous scaffold. Compared with the construction of a cartilage scaffold, the scaffold-free technique is simple and easy to construct tissue-engineered cartilage, avoiding the negative effects such as potential toxicity response of exogenous materials, but the resulting engineered cartilage is limited in mechanical properties, while the mechanical properties of cartilage scaffold can be controlled by adjusting the materials and manufacturing process (Lee et al., 2017; Grogan et al., 2020).

For clinical application, artificial AC made by tissue engineering with and without scaffolds have potential in the clinical treatment of OA and other joint injuries and degeneration. However, the clinical transformation of the tissue engineering-based approaches needs further animal and human clinical trials to ensure the safety and effectiveness of clinical application, which is lacking in some clinical feasible technologies of forming artificial AC (Spencer et al., 2018). Moreover, despite full restoration of chondral structure and function, limitations of artificial grafts like inflammation and life span remain a challenge (Armiento et al., 2018; Zylinska et al., 2018), in which case further surgery may request in follow-up healing procedures. To date, hydrogels-based artificial cartilage demonstrates the best performance among various biomaterials used in tissue engineering (Vega et al., 2017). Though that the clinical translation of the tissue engineering-based approaches is still some time away, with emerging and developing new generation technologies, promising results are foreseen.
